# Clinical correlates of anxiety disorders : Tunisian study about 436 subjects

**DOI:** 10.1192/j.eurpsy.2022.985

**Published:** 2022-09-01

**Authors:** M. Jabeur, L. Gassab, A. Ayadi, B. Ben Mohamed, F. Zaafrane, L. Gaha

**Affiliations:** Research laboratory LR 05 ES 10 “Vulnerability to Psychotic Disorders”, Faculty of medicine, University of Monastir, Psychiatry Department, University Hospital Of Monastir, Monastir, Tunisia

**Keywords:** Anxiety disorders, recurrence, comorbidities, clinical correlates

## Abstract

**Introduction:**

Anxiety disorders are very common and burdensome mental illnesses worldwide, characterized by exagerated feelings of worry and fear. These disorders are highly comorbid with other conditions.

**Objectives:**

The aim of our study is to explore the physical and psychiatric comorbidities and their clinical correlates. The second objective is to identify the predictors of recurrence of anxiety disorders.

**Methods:**

Our study concerned 436 outpatients who met DSM-V diagnostic criteria for anxiety disorders and were followed in the Department of Psychiatry of Monastir (Tunisia) between 1998 and 2017. Selective mutism and seperation anxiety were excluded for lack of cases.

**Results:**

Our results demonstrated that Generalized Anxiety Disorder (GAD) was significantly associated with cardiovascular comorbidity (OR=3.208). Social Anxiety Disorder (SAD) was significantly correlated to avoidant personality disorder (OR=17). Patients with suicide attempts are more likely to have a comorbid personality disorder (OR=11.606). Being married and having a later age of onset are predictors of having comorbid depressive disorder. Furthermore, being married, having an anxiety-anxiety comorbidity and a longer duration of untreated illness (DUI) are predictors of recurrence.

**Conclusions:**

Our study highlights the fact that comorbidities (physical and psychopathological) call for a closer follow up due to the higher risk of recurrence, the higher risk of suicide attempts and the poorer treatment response.

**Disclosure:**

No significant relationships.

